# From Soybean residue to advanced supercapacitors

**DOI:** 10.1038/srep16618

**Published:** 2015-11-16

**Authors:** G. A. Ferrero, A. B. Fuertes, M. Sevilla

**Affiliations:** 1 Instituto Nacional del Carbón (CSIC), P.O. Box 73, Oviedo 33080, Spain

## Abstract

Supercapacitor technology is an extremely timely area of research with fierce international competition to develop cost-effective, environmentally friendlier EC electrode materials that have real world application. Herein, nitrogen-doped carbons with large specific surface area, optimized micropore structure and surface chemistry have been prepared by means of an environmentally sound hydrothermal carbonization process using defatted soybean (*i.e*., *Soybean meal*), a widely available and cost-effective protein-rich biomass, as precursor followed by a chemical activation step. When tested as supercapacitor electrodes in aqueous electrolytes (*i.e.* H_2_SO_4_ and Li_2_SO_4_), they demonstrate excellent capacitive performance and robustness, with high values of specific capacitance in both gravimetric (250–260 and 176 F g^−1^ in H_2_SO_4_ and Li_2_SO_4_ respectively) and volumetric (150–210 and 102 F cm^−3^ in H_2_SO_4_ and Li_2_SO_4_ respectively) units, and remarkable rate capability (>60% capacitance retention at 20 A g^−1^ in both media). Interestingly, when Li_2_SO_4_ is used, the voltage window is extended up to 1.7 V (in contrast to 1.1 V in H_2_SO_4_). Thus, the amount of energy stored is increased by 50% compared to H_2_SO_4_ electrolyte, enabling this environmentally sound Li_2_SO_4_-based supercapacitor to deliver ~12 Wh kg^−1^ at a high power density of ~2 kW kg^−1^.

Electrochemical capacitors (ECs), also known as supercapacitors, represent an attractive energy storage technology for portable electronic devices, cold-starting assistants, electric vehicles, etc., as a result of their better rate capability and longer cyclic life compared to secondary batteries^1^,^2^,^3^,^4^. ECs store energy by charging electrical double layers *via* reversible ion adsorption on the surface of high-surface area electrodes. The electrodes which are the main components in an EC generally consist of porous activated carbon. This material is highly appropriate for this purpose because of its excellent chemical stability, good electrical conductivity, wide availability, environmental friendliness and low cost^5^. More importantly, the porosity of high surface area carbons can be tailored and adjusted to fit the characteristics of the electrolyte used in the ECs^6^. In addition, the capacitance of the carbon materials used as electrodes can be enhanced through pseudocapacitive effects from the incorporation of certain heteroatoms into the carbon framework^7^. For example, nitrogen groups can enhance specific capacitance through additional Faradaic redox reactions combined with the charging of the double-layer^8^,^9^.

Although many different types of carbons have been investigated in recent years, biomass-based carbon has proved to be a very attractive alternative with an excellent performance as electrode in supercapacitors^10^,^11^. Abundant industrial and agricultural wastes, with little or no economic value, can be used as an inexpensive source of carbon for the production of high added-value products. The criterion for selecting the biomass precursor to produce porous carbons is based on its feasibility to generate features such as abundance of microporosity^11^, hierarchical structures^12^,^13^ or a certain specific morphology that offers short ion-diffusion pathways, such as a nanosheet morphology^14^,^15^. Another important aspect to be taken into account is the presence of certain heteroatoms (*i.e.* nitrogen or sulfur) that can be transferred to the carbon products. Accordingly, N-rich biomass products have received increased interest over the past years^16^,^17^,^18^,^19^. In this study, we have focused our attention on the viability of using an abundant residue, defatted soybean (better known as *Soybean meal* or *Soybean oil cake*), as carbon precursor for electrode materials in supercapacitors. This material can be considered as a solid residue, which is generated after the extraction of oil from the soybean. It is widely used as a filler and source of protein in animal diets^20^,^21^. Interestingly, this abundant and low-cost material has a large amount of proteins (around 50%) and, in consequence, it contains an abundance of nitrogen (around 8–10 wt%)^22^. Owing to its high nitrogen content, defatted soybean constitutes a promising precursor for producing N-doped porous carbons.

The heterogeneous structure of biomass and the presence of inorganic impurities are an obstacle to the production of activated carbons with batch-to-batch reproducible properties. Low-temperature hydrothermal carbonization, an inexpensive, environmentally friendly route is a simple way to overcome such shortcomings. The hydrothermal carbonization of biomass is coupled to a chemical activation step in order to transform the hydrochar product into highly porous carbons^23^. The activation step benefits from the structure of hydrochar which is more aromatic than that of the original biomass, resulting in a larger amount of porous carbon (and sometimes holding the key for the successful transformation of biomass into an advanced porous carbon^24^) and a less tortuous pore network owing to the lower burn-off^25^. In this way, highly microporous carbons with a high specific surface area and a well-developed and uniform porosity can be produced^26^,^27^,^28^. In the present work, this synthesis strategy has been adopted to produce N-doped microporous carbons. For this purpose, a N-rich biomass residue (*i.e.* defatted soybean) was selected as carbon precursor. In a first step, the defatted soybean residue was subjected to hydrothermal treatment at a temperature of 200 °C. Next, in order to generate a microporous network, the hydrochar product was chemically activated with KOH at temperatures in the 600–800 °C range. The performance of these microporous carbons as supercapacitor electrodes was tested in acidic (1 M H_2_SO_4_) and more environmentally sound neutral (1 M Li_2_SO_4_) aqueous electrolytes.

## Results

### Synthesis of N-doped porous carbons

**Figure 1:**
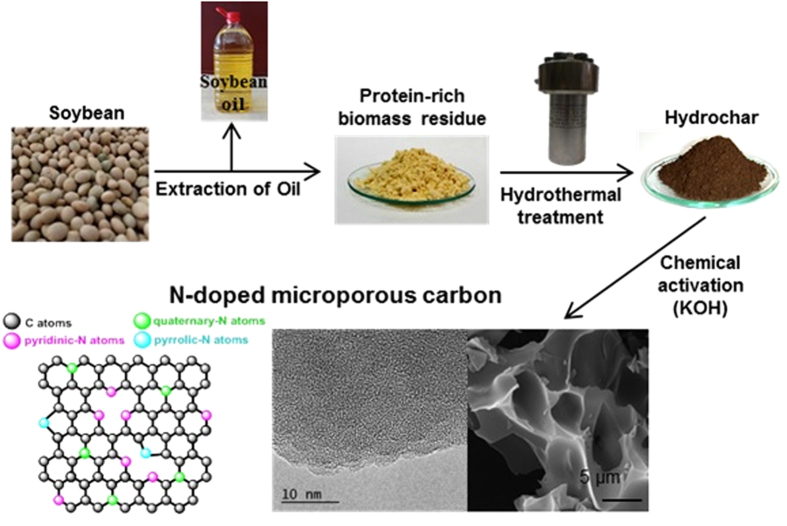
Schematic of the synthesis of N-doped microporous carbon from a protein-rich biomass residue derived from soybean (photos of soybean and protein-rich biomass residue were taken by G. A. Ferrero, photos of hydrochar and SEM and TEM pictures were taken by A. B. Fuertes, photos of HTC reactor and soybean oil were taken by M. Sevilla and the picture of N-moieties was done by M. Sevilla).

Soybean is widely used for oil production, mainly but not entirely, for human consumption. Even though soybean origins are in Southeast Asia, nowadays it is produced worldwide, ensuring easy access. In the last decade, the oil production from soybean has steadily grown, from ~30 million tons in 2004 to more than 45 million tons in 2014^29^,^30^,^31^. This production process leaves behind high amounts of a solid residue, *i.e.* defatted soybean, better known as soybean meal (see Fig. 1). This soybean meal has been traditionally used for animal feed due to its high protein content. However, this high protein content makes this abundant and cost-effective residue highly suitable for a more advanced application, such as its use as precursor for the fabrication of high added-value materials, *i.e.* N-doped porous carbon materials. Herein we demonstrate that this can be achieved based on a “green” process such as hydrothermal carbonization, as depicted in Fig. 1. In this regard, some of us have previously shown that the hydrothermal co-carbonization of glucose with a N-rich biomass of low carbohydrate content (*i.e.* microalgae *Spirulina Platensis*) has beneficial effects on both the hydrochar yield and N content^32^. The same strategy has been followed in this work, resulting in a two-fold increase in the hydrochar yield and a three-fold increase in the nitrogen efficiency (defined as the percentage of N contained in the defatted soybean/glucose mixture retained in the hydrochar), as is shown in Supplementary Fig. S1a online. Furthermore, as can be deduced from Fig. S1b, the degree of carbonization (the higher the degree of carbonization, the lower the H/C and O/C ratios) of the dSB/glucose-derived hydrochar is higher than that of the dSB-derived hydrochar, although both have the same N/C ratio. This result suggests that a higher product yield would be obtained in the activation step as a consequence of a lower burn-off, as has been previously shown when comparing hydrochar with biomass^25^. Indeed, a 10% increase in product yield was recorded. This result is important from a technological and environmental standpoint as it shows that a smaller amount of corrosive KOH can be used to generate the same amount of activated carbon.

### Structural and chemical properties of the porous carbons

**Figure 2:**
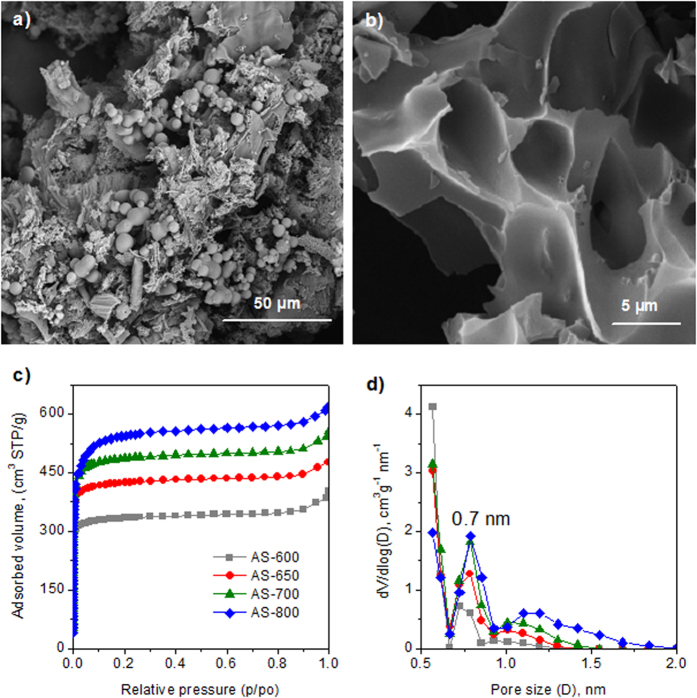
SEM images of the (**a**) dSB/glucose-derived hydrochar, (**b**) porous carbon AS-700, and (**c**) nitrogen sorption isotherms and (**d**) pore size distributions for the AS-600, AS-650, AS-700 and AS-800 porous carbon samples.

The morphology of the hydrochar and activated carbon materials was investigated by means of scanning electron microscopy (SEM). As can be seen in Fig. 1a, the hydrochar sample contains numerous sphere-like microparticles on the surface of larger particles, which are generated as a consequence of the hydrothermal carbonization of glucose and the saccharides present in the defatted soybean residue. On the other hand, the activated samples are made up of particles that have an irregular morphology and large conchoidal cavities, like other hydrochar-derived activated carbons (see Fig. 2b)^24^,^25^,^26^. Even though the material is composed of relatively large particles (5–30 μm, see Supplementary Fig. S2a online), such large conchoidal cavities could ensure that the species gain fast access to the inner pore structure.

A more detailed insight into the porosity of the carbon particles was acquired from nitrogen physisorption at −196 °C. The corresponding nitrogen sorption isotherms are depicted in Fig. 2c. All the carbon materials display a type I isotherm, which is typical of microporous materials, with a large sharp knee for relative pressures <0.1 and a horizontal plateau for higher relative pressures. The slight increase in adsorption recorded at relative pressures higher than 0.9 is attributable to the smallest conchoidal cavities. The highly microporous network is further confirmed by the pore size distributions displayed in Fig. 2d. Thus, the porosity of the samples is made up exclusively of micropores centered at ~0.7 nm, with no pores >2 nm. Interestingly, as the carbonization temperature increases, a second micropore system develops at ~1.1 nm. This second micropore system probably stems from the enlargement of smaller pores already formed during heat-treatment by means of gasification by the CO_2_ evolved in the decomposition of the K_2_CO_3_ generated during the activation process for temperatures higher than 700 °C, according to the reaction: K_2_CO_3_ = 4K_2_O + CO_2_^26^,^33^. The textural properties of these materials are summarized in Table 1. The carbon materials possess high specific surface areas and large pore volumes, which increase with the activation temperature from 1340 m^2^ g^−1^ (AS-600) to 2130 m^2^ g^−1^ (AS-800), and from 0.58 cm^3^ g^−1^ (AS-600) to 0.92 cm^3^ g^−1^ (AS-800) respectively. Importantly, the porosity of these carbons is made up almost exclusively of micropores, the micropore volume comprising more than 90% of the total pore volume.

**Figure 3:**
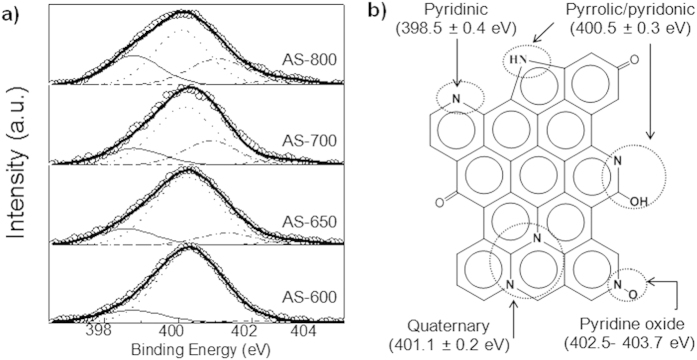
(**a**) XPS high resolution N 1s spectra of the porous carbon samples and (**b**) scheme of the different nitrogen binding motifs identified in the porous carbons.

The bulk chemical composition of defatted soybean and the dSb-derived porous carbons deduced by elemental analysis is shown in Table 1. It can be seen that the activated carbons have a large oxygen content, which decreases with the activation temperature from ~25% at 600 °C to ~14% at 800 °C. More importantly, N-doping is confirmed by values in the range of 1.6–4%. Furthermore, the nitrogen heteroatoms are uniformly distributed throughout the N-doped particles, as can be deduced from the elemental energy-dispersive X-ray (EDX) mapping images in Supplementary Fig. S2b-c online. The chemical nature of the nitrogen groups was examined by XPS analysis. High-resolution N 1s spectra are displayed in Fig. 3a and the relative contribution of each moiety is listed in Supplementary Table S1 online. The samples obtained at T > 600°C exhibit three main peaks that can be assigned to pyridinic-N (398.6 ± 0.2 eV), pyrrolic-/pyridonic-N (400.4 ± 0.2 eV) and quaternary-N (401.1 ± 0.2 eV), and a minor peak that is attributed to pyridine-N-oxides (402.9 ± 0.3 eV)^34^,^35^. The identified binding motifs are depicted in Fig. 3b. A clear decrease in the number of less stable species, *i.e.* pyrrolic-/pyridonic-N, and an increase in the amount of more stable ones, *i.e.* pyridinic- and quaternary-N, is recorded with the rise in synthesis temperature. Thus, AS-800 contains almost half of the content of pyrrolic nitrogen of AS-600. It has been shown that pseudocapacitive interactions take place on the negatively charged pyrrolic-N and pyridinic-N groups, while the positive charge on quaternary-N and pyridine-N-oxides favours the electron transfer through the carbon, enhancing the conductivity of the carbon materials^36^,^37^. The removal of oxygen functionalities, coupled to the increase in N-Q and N-X and carbon ordering with the rise in carbonization temperature, explains the enhancement of electronic conductivity as the synthesis temperature increases (see Table 1).

### Capacitive performance in aqueous electrolytes

**Figure 4:**
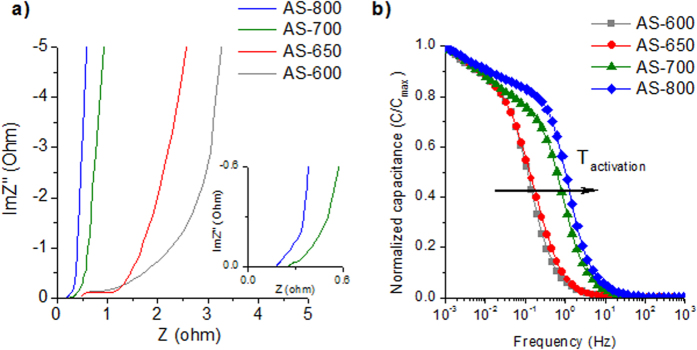
(**a**) Nyquist plot and (**b**) frequency response for the N-doped porous carbons. Electrolyte: 1 M H_2_SO_4_.

Because of the microporous nature of these materials and the abundance of nitrogen and oxygen functionalities, their capacitive behavior was tested in aqueous electrolytes, *i.e.* H_2_SO_4_ and Li_2_SO_4_. Figure 4 shows the results obtained from EIS measurements for 1 M H_2_SO_4_ electrolyte. The Nyquist plots in Fig. 4a show a decrease in the equivalent series resistance (ESR) with the rise in synthesis temperature, in agreement with the enhancement of the electronic conductivity registered with the rise in synthesis temperature (from 6·10^−3^ S cm^−1^ at 600 °C up to 2.5 S cm^−1^ at 800 °C, Table 1). Furthermore, a clear semicircle associated with charge transfer processes is detected in AS-600 and AS-650, indicating the presence of pseudocapacitance phenomena contributed by the nitrogen and oxygen functionalities. Similarly, a small semicircle is registered in AS-700 as can be seen in the inset of Fig. 4a. This feature is lacking in AS-800, suggesting the absence of pseudocapacitance phenomena. These results are in agreement with the cyclic voltammograms (CV) in Supplementary Fig. S3 online. Thus, the CV curves corresponding to the materials synthesized at T ≤ 700 °C are quasi-rectangular, with a clear faradaic hump at cell voltages < 0.4–0.5 V (a detailed analysis of the behavior of the positive and negative electrodes in the supercapacitor can be found in the Supporting Information in Supplementary Fig. S4 online). In contrast, sample AS-800 shows a rectangular shape typical of an electrochemical double-layer capacitor (see Supplementary Fig. S3d online). The variation of the normalized capacitance with frequency is represented in Fig. 4b. A faster frequency response can be clearly seen with the increase in activation temperature. Accordingly, AS-800 and AS-700 have small relaxation time constants of 1.4 s and 1.6 s respectively, which are considerably smaller values than those of the samples synthesized at lower temperatures (*i.e.* 8.0 s for AS-650 and 8.6 s for AS-600). The diminution of the relaxation time constant with the rise in activation temperature can be ascribed to: i) better ion transport characteristics owing to the development of a second micropore system centered at 1.1 nm; ii) an enhancement of the electronic conductivity (see Table 1), iii) the removal of oxygen functionalities which may interact with the ion solvated shells, slowing down the motion of ions^38^,^39^, and iv) the diminishing impact of slow redox reactions (pseudocapacitance). Consequently, the rate performance increases with the activation temperature, which is confirmed by the CV curves in Supplementary Fig. S3 online. Indeed, in the case of AS-800 and AS-700 the CV shape is maintained up to 200 mV s^−1^ with a good capacitance retention (72% for AS-800 and 62% for AS-700). On the other hand, AS-650 and AS-600 are only able to work up to 50 mV s^−1^ and they display a lower capacitance retention (~64%).

**Figure 5:**
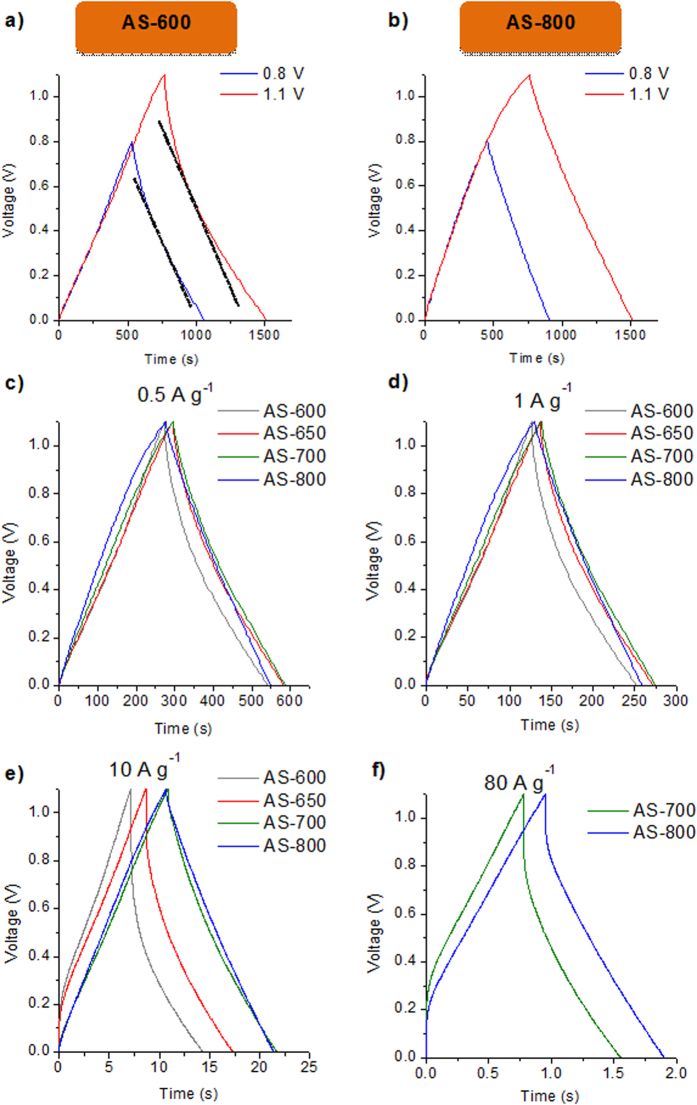
Enlargement of the voltage window evaluated by CD at 0.2 A g^−1^ for (**a**) AS-600 and (**b**) AS-800. CD voltage profiles at (**c**) 0.5 A g^−1^, (**d**) 1 A g^−1^, (**e**) 10 A g^−1^ and (**f**) 80 A g^−1^ for the N-doped microporous carbon materials. Electrolyte: H_2_SO_4_.

Constant current charge/discharge cycling (CD) experiments were performed in 1M H_2_SO_4_ electrolyte at current densities in the 0.1–80 A g^−1^ range. Taking into account the fact that heteroatom-doped carbon materials normally exhibit high resistance towards corrosion^40^,^41^, an enlargement of the voltage window up to 1.1 V was first explored. Figure 5b and Supplementary S5 online show the voltage profiles obtained in the CD test at 0.2 A g^−1^ as the cell voltage was increased from 0.8 V to 1.1 V for the N-doped microporous carbons. Irrespective of the cell voltage used, the voltage profiles are symmetrical for all the materials, with coulombic efficiencies >97%, indicating that no secondary reactions, such as electrolyte decomposition or irreversible carbon corrosion, are taking place. This is corroborated by the results in Supplementary Fig. S4 online. Further confirmation of the stability of the supercapacitors at 1.1 V was obtained by long-term CD at 5 A g^−1^. Thus, as shown in Supplementary Fig. S6 online, capacitance loss after 10000 cycles is lower than 10% for all the N-doped microporous carbons. On the other hand, the voltage profiles for a cell voltage of 0.8 V are curved for AS-600 (Fig. 5a), AS-650 (Supplementary Fig. S5a online) and AS-700 (Supplementary Fig. S5b online), but linear for AS-800 (Fig. 5b). These results reveal that the contribution of pseudocapacitance to electrode performance decays with the rise in activation temperature, which is in accordance with the diminution of the nitrogen (and decreasing presence of N-5 and N-6) and oxygen contents. This deviation from linearity increases with cell voltage (including AS-800), hinting at an increase in the contribution of pseudocapacitance to the energy storage process, with a consequent enhancement of the specific capacitance of *ca.* 20%. The voltage profiles of the carbon materials are compared at diverse current densities in Fig. 5c–f. The small IR drop registered in AS-700 and AS-800 regardless of the discharge rate reflects a small equivalent series resistance (ESR) in the supercapacitor, which agrees well with the EIS measurements, and makes it possible to attain discharge rates of 80 A g^−1^. AS-600 and AS-650, on the other hand, cannot withstand discharge rates higher than 20 A g^−1^. Table 2 summarizes the values of specific capacitance obtained for the microporous materials at a cell voltage of 1.1 V and at current density of 0.2 A g^−1^. All the materials exhibit similar values of specific capacitance, in the ~ 250–260 F g^−1^ range, independently of the value of surface area, which reflects the increasing contribution of pseudocapacitance with the decrease in activation temperature. Thus, for the AS-600 sample, the material with the highest N and O contents (see Table 1), the surface area-normalized capacitance is *ca.* 19 μF cm^−2^, which is a considerably higher value than that obtained for the AS-800 sample (12 μF cm^−2^), the material that has the lowest N and O contents (see Table 1). These values of specific capacitance are comparable, or superior, to some types of carbon reported in the literature such as carbon derived from seaweeds, different graphene materials, carbon spheres, carbon nanocages, carbon nanofibers, activated carbons, or hydrochar-based porous carbons. A comparative table of these materials is provided in the Supporting Information (Supplementary Table S2 online).

**Figure 6:**
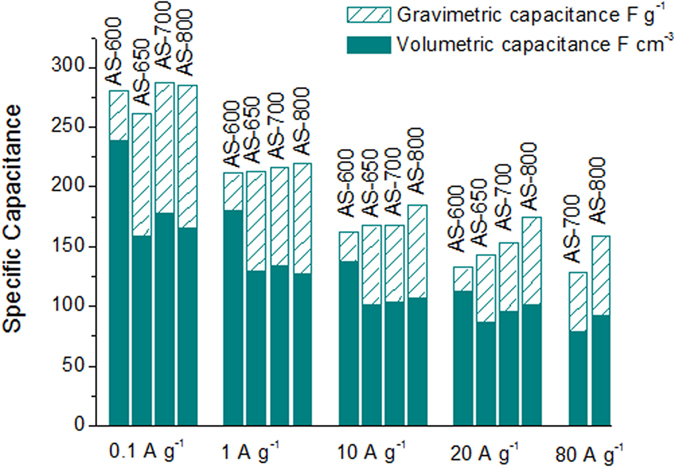
Variation of gravimetric and volumetric specific capacitance with increasing current density for the N-doped porous carbons in 1 M H_2_SO_4_ (Voltage window: 1.1 V).

Generally, porous carbons used in supercapacitors possess low packing densities, usually <0.5 g cm^−3^ 42. This means that, in spite of their high specific gravimetric capacitance, many of these materials undergo a significant reduction in volumetric performance that makes them less competitive for portable, compact energy storage systems^43^,^44^,^45^. However, the density of the electrodes fabricated with the microporous carbons developed in this work is relatively high, in the 0.58 to 0.85 g cm^−3^ range, which leads to high volumetric capacitances in the 150–210 F cm^−3^ range at 0.2 A g^−1^ (see Table 2), values which compare very well with those of other materials reported in the literature (see Supplementary Fig. S7 online). In addition, these activated carbons show relatively high rate capabilities, as can be deduced from Fig. 6. Thus, the samples obtained at low activation temperatures (*i.e.* AS-600 and AS-650) can withstand current densities of up to 20 A g^−1^ with ~50% of capacitance fading. More importantly, the AS-700 and AS-800 samples are able to maintain good capacitance values of *ca.* 140 F g^−1^ (87 F cm^−3^) and 170 F g^−1^ (99 F cm^−3^) respectively at a high current density of 40 A g^−1^, as well as remarkable capacitance retentions of 45 and 56% respectively for a 400-fold discharge rate increase from 0.2 to 80 A g^−1^. Thus, if both gravimetric and volumetric performances are taken into consideration, the carbon synthesized at the lowest temperature (*i.e.* AS-600) would appear the most appropriate as a supercapacitor electrode for low to moderate discharge rates (<5 A g^−1^), whereas the highest temperature carbon AS-800 would be more suitable for high rates. It is clear from Supplementary Table S2 online that the activated carbons developed in this work compare favorably with high-performing EC materials reported so far in the literature, including graphene/graphene-like materials or advanced activated carbons.

As the energy density of an EC only increases linearly with capacitance, but quadratically with the cell voltage (see Equation 4 in the Experimental Section), a great deal of research nowadays is focused on increasing the EC cell voltage by using electrolytes with larger electrochemical stability windows. Thermodinamically, the working voltage window in an aqueous electrolyte is limited by the decomposition of the water, *i.e.* 1.23 V. However, in practice, for acidic and alkaline electrolytes, the working voltage is reduced to values below 1 V (except in the case of materials with a rich surface chemistry like the ones synthesized here) owing to carbon oxidation in the positive electrode^41^ or hydrogen evolution in the negative electrode^40^. On the contrary, when the pH of the electrolyte solution is around 7 (neutral conditions), the operating voltage can be increased to above 1.23 V as a result of large overpotentials for water decomposition (which vary depending on the type of carbon used), especially in the negative electrode where hydrogen electrosorption takes place^46^,^47^,^48^,^49^. This feature has spurred interest in neutral electrolytes as they allow high energy densities, with the additional benefits of being environmentally friendly and non-corrosive. In this work, the use of Li_2_SO_4_ as neutral electrolyte was studied owing to its high solubility^50^. For the analysis of electrochemical performance in Li_2_SO_4_, the AS-800 sample was selected due to its high specific surface area, optimized pore system for ion storage (pore system of size ~0.7 nm) and transport (pore system of size ~ 1.1 nm), and good electronic conductivity (see Table 1).

**Figure 7:**
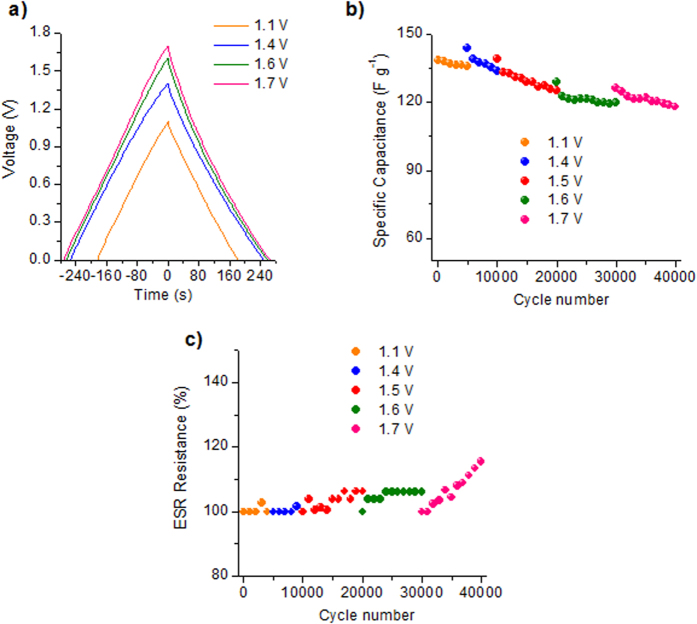
(**a**) Enlargement of the voltage window evaluated by CD at 0.2 A g^−1^, (**b**) long-term stability at different cell voltages in the 1.1–1.7 V range evaluated by charge-discharge at a constant current of 5 A g^−1^ and (**c**) ESR resistance variation ratio (R_n_/R_1_ **× **100, where R_n_ is the resistance at the n^th^ cycle and R_1_ at the 1^st^ cycle) at different cell voltages in the 1.1–1.7 V range evaluated by charge-discharge at a constant current of 5 A g^−1^ for the microporous materials in 1 M Li_2_SO_4_ electrolyte.

Figure 7a shows the galvanostatic charge-discharge curves corresponding to the AS-800-based symmetrical supercapacitor in 1 M Li_2_SO_4_ electrolyte at a low discharge rate of 0.2 A g^−1^ with a voltage window that increases up to 1.7 V. Independently of the cell voltage, the coulombic efficiency is higher than 96%, indicating ideal capacitive behavior. The stability of the symmetric supercapacitor at different voltage windows was assessed through CD at a constant current of 5 A g^−1^ over 5000–10000 cycles at each cell voltage. As can be seen in Fig. 7b, capacitance fading is lower than 10% regardless of the cell voltage, suggesting a good stability. However, apart from capacitance fading, another important parameter to be taken into account when assesing the possibility of failure of a supercapacitor is the increase in ESR resistance, which is shown in Fig. 7c. As can be seen, cell resistance remains almost constant for cell voltages <1.6 V. However, at 1.7 V, the cell resistance steadily increases, although the increase over 10000 cycles is much lower than the end-of-life value established by manufacturers, *i.e.* an increase by 100%^51^. It can therefore be affirmed that the supercapacitor is stable up to a cell voltage of 1.7 V. This increase in the working voltage range can be attributed to hydrogen electrosorption in the negative electrode and the non-destructive oxidation of the positive electrode. Thus, as shown in Supplementary Fig. S8 online, hydrogen electrosorption takes place in the negative electrode below 1 V vs. SME (*i.e.* when the cell voltage is >1.3 V) and the positive electrode is oxidized for potentials above the limit of water oxidation, *i.e.* 0.23 V vs. SME. Thereby, the initial enhancement of capacitance observed for cell voltages >1.3 V in Fig. 7b can be ascribed to the dual factor of hydrogen electrosorption and carbon oxidation, as we have previously observed for N-doped activated carbons derived from mixtures of hydrochar and melamine^49^. It is worth noting that the electro-oxidation of the positive electrode leads to a reversible hump at ~−0.1 V *vs.* SME, which is ascribable to redox reactions involving the quinone/hydroquinone pair^40^,^52^,^53^. Hence, the oxidation is not destructive -up to a cell voltage of 1.7 V-, which agrees with the good cycling stability measured in a common two-electrode cell (see Fig. 7b), but it does lead to an increase in ESR resistance (see Fig. 7c) owing to the oxidation of the positive current collector (the evidence for which was clearly obtained when disassembling the cell), which undermines the electrode-current collector contact. However, deterioration of the electrode-collector contact due to some evolution of gases may not be discarded.

**Figure 8:**
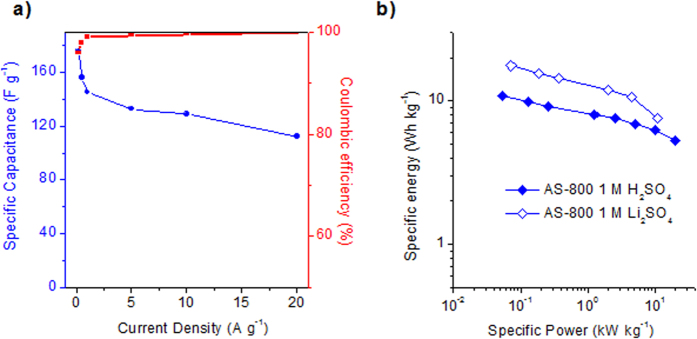
Variation of specific capacitance and coulombic efficiency with increasing current density in 1 M Li_2_SO_4_, and (**b**) Ragone plot for the AS-800 sample in 1 M H_2_SO_4_ (Voltage range: 1.1 V) and 1 M Li_2_SO_4_ (Voltage range: 1.7 V).

As for the H_2_SO_4_ electrolyte, the specific capacitance and rate capability of the supercapacitor in Li_2_SO_4_ was determined from constant current charge-discharge experiments. The variation in specific capacitance with increasing current density is displayed in Fig. 8a, along with the values of coulombic efficiency, which show ideal capacitive behavior regardless of the discharge rate. A specific capacitance of 176 F g^−1^ (102 F cm^−3^) is achieved at 0.2 A g^−1^, which is higher than that of commercial activated carbons in 1 M Li_2_SO_4_ electrolyte^48^,^50^,^54^. Although this value is somewhat lower than that obtained in H_2_SO_4_ electrolyte (*i.e.* 258 F g^−1^), the fact that in Li_2_SO_4_ the EC system can operate at 1.7 V (*vs.* 1.1 V in 1 M H_2_SO_4_) is a clear advantage that compensates for the lower capacitance values relative to the amount of energy stored, as will be discussed below. Furthermore, AS-800 has a specific capacitance of ~130 F g^−1^ (75 F cm^−3^) at a relatively high current density of 10 A g^−1^, which implies a capacitance retention of 74%, a value still superior to that of other activated carbons found in the literature^47^,^48^,^55^. This enhanced rate capability can be ascribed to the engineered surface chemistry and pore structure, which offer a smooth ion diffusion, coupled to a good electronic conductivity. The rate capability was further confirmed by electrochemical impedance spectroscopy (EIS) measurements. It was found that the microporous carbon material possesses a relaxation time constant close to 8 s, as can be deduced from the frequency response of capacitance in Supplementary Fig. S9a online. This value is considerably higher than that found in 1 M H_2_SO_4_ electrolyte, in agreement with the better rate capability recorded in 1 M H_2_SO_4_ compared to 1 M Li_2_SO_4_. On the basis of the EIS analysis, this difference in rate capability can be ascribed to the greater resistance of the supercapacitor (both ESR and EDR) in Li_2_SO_4_ than in H_2_SO_4_. Thus, ESR resistance increases from 0.17 Ohm in H_2_SO_4_ to 0.53 Ohm in Li_2_SO_4_, and EDR resistance (*i.e.* the resistance the electrolyte ions confront when they penetrate the porous structure) increases greatly from 0.17 Ohm in H_2_SO_4_ to 2.6 Ohm in Li_2_SO_4_ (see Supplementary Fig. S9b online). These EDR values are similar to those of other highly microporous biomass-based carbons, such as those derived from tobacco stems^56^, or sawdust/glucose-derived hydrochars^49^, but much higher than that of micro-mesoporous activated carbons (*i.e.* 0.5 Ohm)^49^, revealing hindered ion diffusion through the micropores in the case of the low conductive Li_2_SO_4_.

The energy and power characteristics of the AS-800 sample are represented in the Ragone plot in Fig. 8b. It can be seen that in 1 M H_2_SO_4_, the microporous sample provides a maximum energy density of ~12 Wh kg^−1^/~7 Wh L^−1^ (0.025 kW kg^−1^/0.015 kW L^−1^). The superior rate capability of the AS-800 sample (*vide supra*) is reflected in just a small trade-off between energy and power, so that it is still able to store ~5 Wh kg^−1^/~3 Wh L^−1^ at a high power density of 20 kW kg^−1^/11.6 kW L^−1^. Supplementary Figure S10 online shows a Ragone plot that compares the AS-800 and AS-650 carbon materials with state-of-the-art carbons, including graphene materials, biomass-derived carbons, carbon nanocages, carbon nanofibers, or hierarchical nitrogen-doped carbon. The figure evidences that the synthesized carbon materials provide a performance comparable, or even superior, to many advanced, biomass-based carbon materials found in the literature. More importantly, Fig. 8b shows that the Li_2_SO_4_-based supercapacitor outperforms the H_2_SO_4_-based one in spite of the lower capacitance values achieved thanks to its large working voltage, *i.e.* 1.7 V. Indeed, a maximum specific energy of ~18 Wh kg^−1^/10 Wh L^−1^ is achieved at a power density of ~0.07 kW kg^−1^/0.04 kW L^−1^, which implies a 50% improvement in the amount of energy stored compared to the H_2_SO_4_-based supercapacitor. Furthermore, despite the inferior rate capability registered in Li_2_SO_4_ when compared to H_2_SO_4_, the Li_2_SO_4_-based supercapacitor is able to work up to 10 kW kg^−1^/6 kW L^−1^ delivering 7.5 Wh kg^−1^/4.4 Wh L^−1^. Figure S11 shows a Ragone Plot that compares the energy and power characteristics of AS-800 in 1 M Li_2_SO_4_ with those of different carbon materials in alkali sulfate electrolytes. This figure clearly evidences the superior performance of the microporous carbon developed in the present work as a result of its optimized pore structure and surface chemistry.

## Discussion

High-added value materials, *i.e.* N-doped highly microporous carbon materials with an optimal capacitive performance, have been successfully synthesized from an abundant and low-value agricultural by-product, defatted soybean (*i.e. Soybean meal*). These microporous carbons were obtained by means of a procedure that involves two key steps: i) hydrothermal carbonization for improving the characteristics of the biomass for use as a precursor and ii) chemical activation to generate an appropriate pore structure. The carbon materials thus synthesized have a high specific surface area (up to ~2100 m^2^ g^−1^), a narrow micropore size distribution and a nitrogen content in the 1.6–4 wt% range. The abundance of nitrogen and oxygen functionalities combined with large specific surface areas ascribed to microporosity led to high values of specific capacitance in 1 M H_2_SO_4_ of ~250–260 F g^−1^ at low discharge rates. Furthermore, the relatively high packing density offered by these materials gave rise to remarkable volumetric capacitances in the 150–210 F cm^−3^ range, which is essential for space-constrained applications. By controlling the activation temperature, a material with optimized ion storage (pore system of size ~0.7 nm) and ion/electron transport properties (pore system of size 1.1 nm and electronic conductivity of 2.54 S cm*–1*) was obtained. This material was thus able to retain *ca.* 60% of its capacitance (160 F g^−1^/93 F cm^−3^) at a high rate of 80 A g^−1^ in H_2_SO_4_. The engineered surface chemistry and pore structure of the material also made it possible for this material to work at 20 A g^−1^ with a capacitance retention of 64% in a low conductive electrolyte Li_2_SO_4_. Also worth noting is the large cell voltage achieved in Li_2_SO_4_ (1.7 V), compared to that obtained in H_2_SO_4_ (1.1 V). This large cell voltage compensated by far for the lower specific capacitance of the Li_2_SO_4_-based supercapacitor (*i.e.* 176 F g^−1^/102 F cm^−3^) and, in this way, the amount of energy stored (the key parameter to be enhanced) was 50% greater in Li_2_SO_4_ than in H_2_SO_4_. This environmentally friendly Li_2_SO_4_-based supercapacitor was thus able to deliver ~12 Wh kg^−1^ (7 Wh L^−1^) at a high power density of ~2 kW kg^−1^ (1.2 kW L^−1^). In addition, both the H_2_SO_4_ and Li_2_SO_4_-based supercapacitors showed a good cycling stability under such high cell voltages, with a capacitance retention in the 90–95% range over 10000 cycles of charge-discharge in both cases.

## Methods

### Synthesis of Materials

A defatted soybean material was prepared in the laboratory by means of soxhlet extraction of commercial soya flour with hexane over a period of 17 hours. Afterwards, the defatted soybean solid residue (hereafter denoted as *dSB*) was mixed with glucose and subjected to hydrothermal carbonization. The addition of glucose is necessary to enhance the fixation of nitrogen and hydrochar yield^32^. Briefly, an aqueous solution/dispersion of defatted soybean and glucose (weight ratio of dSB/glucose ~2) was placed in a stainless steel autoclave and heat-treated at 200 °C overnight (15 h). The resulting solid (hydrochar) was recovered by filtration, washed with distilled water, Soxhlet-extracted with acetone and dried at 120 °C.

The hydrochar materials were chemically activated using potassium hydroxide (Sigma-Aldrich). Briefly, a hydrochar sample was thoroughly ground with KOH (KOH/Hydrochar weight ratio of 2). Subsequently, the mixture was heat-treated up to the desired carbonization temperature (*i.e*. 600, 650, 700 or 800 °C) for 1 h, under a nitrogen gas flow. Finally, the solid residue was washed three times with HCl (10%) to remove the potassium compounds and then with distilled water. The carbon particles were collected by centrifugation and dried at 120 °C for several hours. The activated carbons thus synthesized were denoted as *AS–X, X* being the carbonization temperature in °C.

### Characterization

The nitrogen sorption isotherms of the carbon samples were measured at −196 °C using a Micromeritics ASAP 2020 sorptometer. The apparent surface area (S_BET_) was calculated from the N_2_ isotherms using the Brunauer-Emmett-Teller (BET) method. An appropriate relative pressure range was selected to ensure that a positive line intersect of multipoint BET fitting (C > 0) would be obtained and V_ads_(1 − p/p_o_) would increase with p/p_o_^57^,^58^. The total pore volume (V_p_) was determined from the amount of nitrogen adsorbed at a relative pressure (p/p_o_) of 0.95. The micropore volume (V_m_) and the micropore size distributions were determined by means of the quenched-solid density functional theory (QSDFT) method applied to the nitrogen adsorption data and assuming a slit pore model. Scanning electron microscopy (SEM) images were obtained on a Quanta FEG650 (FEI) instrument. X-ray photoelectron spectroscopy (XPS) was carried out on a Specs spectrometer, using Mg K α (1253.6 eV) radiation from a double anode at 150 W. Binding energies for the high resolution spectra were calibrated by setting C 1s to 284.6 eV. Bulk elemental analysis (C, H, N and O) of the samples was carried out on a LECO CHN-932 microanalyzer.

The dc electrical conductivity of the carbon powders was determined in a home-made apparatus (four-probe method) by pressing the carbon powders between two plungers into a hollow Nylon cylinder (inner diameter of 8 mm), and applying a pressure of 7.1 MPa.

### Electrochemical measurements

Electrodes were prepared by mixing 85 wt% of active material, 10 wt % of polytetrafluoroethylene (PTFE) binder (Aldrich, 60 wt% suspension in water) and 5 wt% of the conductive additive Super C65 (Timcal company). The electrochemical measurements were performed in two-electrode Swagelok type cells. Electrochemical capacitors were assembled using two carbon electrodes of similar mass (carbon load per electrode ~5–6 mg cm^−2^) and thickness (~100–150 μm), electrically insulated by a glassy fibrous separator. Stainless steel and gold current collectors were used with 1 M Li_2_SO_4_ and 1 M H_2_SO_4_ electrolytes, respectively. To evaluate the polarization characteristics of the positive and the negative electrode independently, a special two-electrode cell provided with a reference electrode Hg/Hg_2_SO_4_ (SME, saturated) was employed. The electrochemical characterization was performed using a computer controlled potentiostat (Biologic VMP3 multichannel generator) and consisted of cyclic voltammetry, galvanostatic charge-discharge cycling experiments and electrochemical impedance spectroscopy measurements.

Cyclic voltammetry experiments (CVs) were performed in the 0–1.1 V in the H_2_SO_4_ medium and in the 0–1.7 V range in the Li_2_SO_4_ medium at increasing sweep rates from 1 to 200 mV s^−1^. Plots of differential capacitance *vs.* voltage were drawn using the formula:


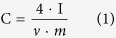


where I = current (A), ʋ = scan rate (V s^−1^) and m = mass (grams) of carbon material in the supercapacitor.

Constant current charge/discharge tests were performed in the 0–1.1 V range in the H_2_SO_4_ medium and in the 0–1.7 V range in the Li_2_SO_4_ medium at current densities in the 0.1 to 80 A g^−1^ range (on the basis of the active mass of a single electrode). The specific gravimetric capacitance of a single electrode (F g^−1^) determined from the galvanostatic cycles was calculated by means of the formula


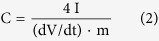


where dV/dt = slope of the discharge curve (V s^−1^).

Taking into account the dependence of specific capacitance on the voltage in the present materials due to the presence of pseudocapacitance, the selection of an appropriate voltage range for the determination of the slope is very important to avoid overestimating the specific capacitance. As most supercapacitors are operated in the range of V_max_ to approximately ½ V_max_, the upper half of the discharge curve was used to determine the slope of the discharge curve^59^. As an example, for AS-600, the value of specific capacitance thus determined in H_2_SO_4_ is 249 F g^−1^, compared to a value >300 F g^−1^ that would be obtained if the lower half of the voltage range were included in the calculations.

Electrochemical impedance spectroscopy (EIS) was carried out in the as-assembled supercapacitors at open-circuit voltage (*i.e*., 0 V) within the frequency range of 1 mHz to 100 kHz and a 10 mV AC amplitude. Nyquist plots and Bode plots of the dependence of capacitance on frequency were recorded to characterize the impedance of the tested samples. The specific gravimetric capacitance of a single electrode, C_EIS_ (F/g), was calculated from the following formula and normalized with respect to the highest specific gravimetric capacitance, *i.e.* the specific gravimetric capacitance at the lowest frequency (1 mHz):


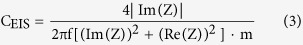


where *f* is the operating frequency (Hz), and *Im(Z)* and *Re(Z)* are the imaginary and real components of the total device resistance (Ohm). The relaxation time constant, τ_0_, which defines the boundary between the regions of capacitive and resistive behaviors of the supercapacitor, was deduced from the frequency *f*_*0*_ as follows: τ_0_ = 1/*f*_*0*_, where *f*_*0*_ can be obtained from the real capacitance plot at 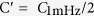. The equivalent series resistance (ESR) was determined from the intercept of the Nyquist plot with the real impedance axis at the highest frequency, whereas the equivalent distributed resistance (EDR) was calculated from the linear projection of the vertical portion at low frequencies to the real axis (after the subtraction of ESR and the resistance of electrode/current collector interface, *i.e.* R_i_)^60^.

The constant current charge/discharge tests were used to determine the gravimetric energy (in Wh kg^−1^) and power (in kW kg^−1^) densities of the supercapacitor cells at different discharge rates based on the following formulae:


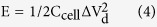



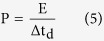


where C_cell_ is the specific capacitance of the total cell (in F g^−1^), ΔV_d_ is the operation voltage (V_max_ – IR_drop_), and Δt_d_ is the discharge time.

For the calculation of specific volumetric capacitance, and volumetric energy and power densities, the density of the electrode was used, this being determined from its thickness (measured with a micrometer caliper) and area (0.785 cm^2^).

## Additional Information

**How to cite this article**: Ferrero, G.A. *et al.* From Soybean residue to advanced supercapacitors. *Sci. Rep.* **5**, 16618; doi: 10.1038/srep16618 (2015).

## Supplementary Material

Supplementary Information

## Figures and Tables

**Table 1:** Physico-chemical properties of the microporous carbon samples.

Sample Code	Textural properties			Chemical Composition (wt %)			Electronic conductivity (S cm^−1^)
	S_BET_ (m^2^ g^−1^)	V_p_(cm^3^ g^−1^)^a^	V_mi_(cm^3^ g^−1^)^b^	N	O	C	
dSB	−	−	−	8.1	40.8	44.7	−
AS-600	1340	0.58	0.53	4.0	24.6	68.3	0.006
AS-650	1710	0.72	0.67	2.6	20.9	74.3	0.066
AS-700	1950	0.82	0.77	1.6	17.2	80.0	0.931
AS-800	2130	0.92	0.87	1.6	13.9	84.1	2.54

^a^Pore volume determined at p/p_0_ = 0.95.

^b^The micropore volume was obtained by the QSDFT method.

**Table 2:** Specific, volumetric and surface area-normalized capacitances calculated at 0.2 A g^−1^ at a voltage window of 1.1 V.

Sample Code	Electrode density (g cm^−3^)	Specific capacitance (F g^−1^)	Volumetric capacitance (F cm^−3^)	Surface area-normalized capacitance (μF cm^−2^)
AS-600	0.85	249	212	18.6
AS-650	0.61	254	157	14.9
AS-700	0.62	261	159	13.4
AS-800	0.58	258	150	12.1

Electrolyte: 1 M H2SO4
