# Supplementary material for: From Soybean residue to advanced supercapacitors
Source: Sci Rep. 2015 Nov 16;5:16618. doi: 10.1038/srep16618 (PMC4645100; doi:10.1038/srep16618)
Supplement: Supplementary Information [file srep16618-s1.pdf]

Supporting information

From Soybean residue to advanced supercapacitors

G.A. Ferrero, A. B. Fuertes, M. Sevilla*

Instituto Nacional del Carbón (CSIC), P.O. Box 73, Oviedo 33080, Spain

* Corresponding author: martasev@incar.csic.es

Table S1. Relative amounts of the different nitrogen functional groups, as deduced by XPS measurements.

Sample Code	Nitrogen functional groups (%)			
	Pyridinic N (N-6)	Pyrrolic N/pyridonic-N (N-5)	Quaternary N (N-Q)	Pyridine-N-oxide (N-O)
**AS-600**	13.9	83.9	-	2.2
**AS-650**	15	70.3	11.2	3.5
**AS-700**	15.8	58.3	21.3	4.5
**AS-800**	25.1	46.7	23.7	4.5

Table S2. Comparison of the gravimetric specific capacitance of various carbon materials in aqueous electrolyte using a two-electrode cell.

Material	Electrolyte	Gravimetric capacitance (F g^{-1})			Ref.
		Low discharge rates	Discharge rate 1 A g^{-1}	High discharge rates	
AS-650	1 M H_2SO_4	254 (0.2 A g^{-1})	213	~ 143 (20 A g^{-1})	This work
AS-700	1 M H_2SO_4	261 (0.2 A g^{-1})	216	~ 128 (80 A g^{-1})	This work
AS-800	1 M H_2SO_4	258 (0.2 A g^{-1})	220	~ 159 (80 A g^{-1})	This work
LN-600 (N-doped carbon)	1 M H_2SO_4	264 (0.05 A g^{-1})	-	-	1
3D-GO	6 M KOH	-	282	~ 165 (33 A g^{-1})	2
Graphene-like nanosheets	6 M KOH	-	268	185 (30 A g^{-1})	3
High density porous graphene macroform	6 M KOH	238 (0.1 A g^{-1})	200	~ 181 (20 A g^{-1})	4
N-carbon spheres	1 M H_2SO_4	190 (0.05 A g^{-1})	-	~120 (20 A g^{-1})	5
Carbon nanocages	6 M KOH	-	248	~ 185 (100 A g^{-1})	6
Carbon nanofibers	6 M KOH	-	202	~165 (30 A g^{-1})	7
Activated carbon nanoplates	1 M H_2SO_4	-	264	~ 120 (52.5 A g^{-1})	8
O- and N-doped activated carbon	1 M H_2SO_4	368 (0.05 A g^{-1})	-	-	9
Hydrochar-based porous carbons	1 M H_2SO_4	270-320 (0.1 A g^{-1})	230-260	~ 180-190 (100 A g^{-1})	10

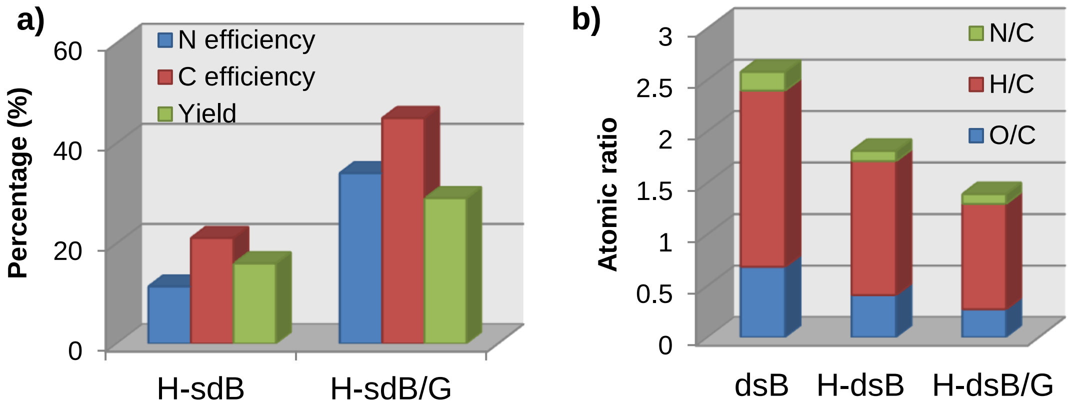

Figure S1. (a) Nitrogen and carbon yields and (b) atomic ratios of nitrogen, hydrogen and oxygen *versus* carbon for defatted soybean (dSB), defatted soybean-derived hydrochar (H-dSB), and defatted soybean/glucose-derived hydrochar (H-dSB/G).

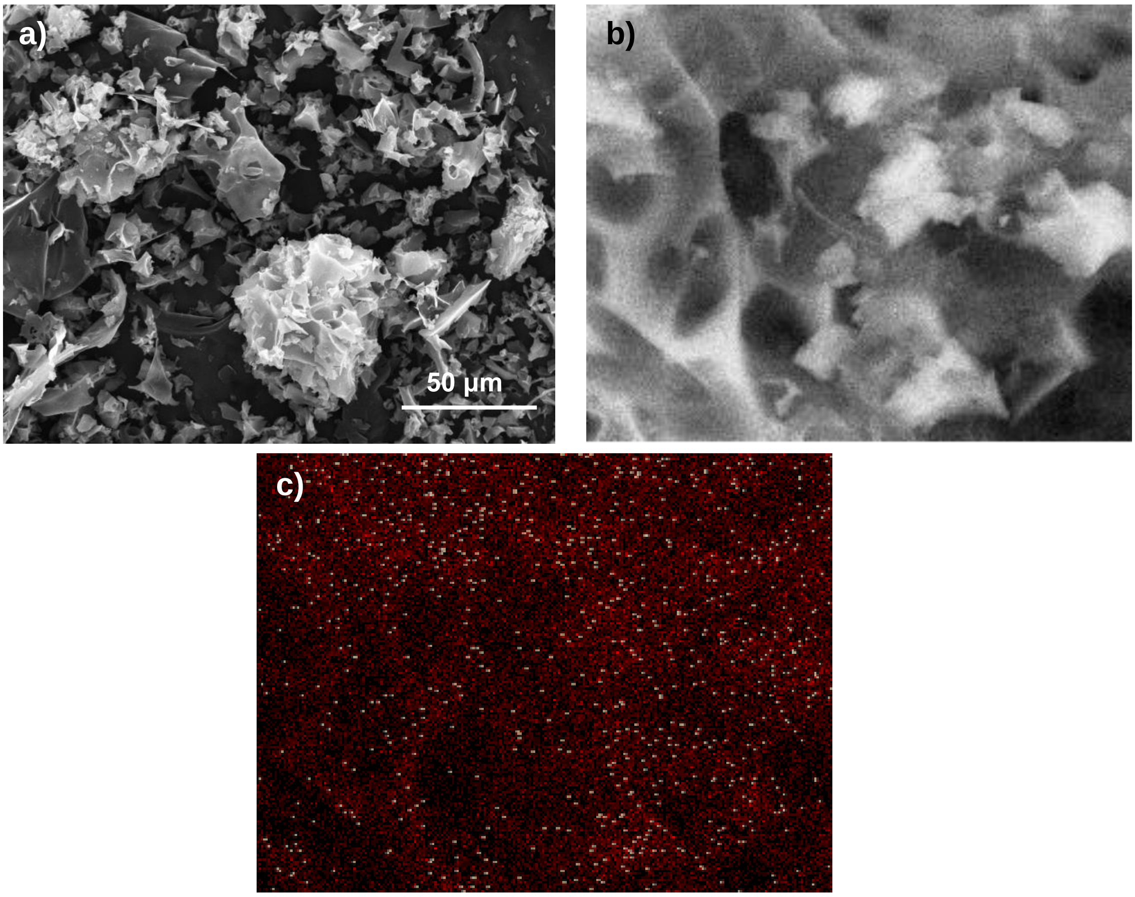

Figure S2. (a) SEM image of AS-700, (b) high-magnification image of AS-600 and (c) its corresponding EDX mappings for carbon (red) and nitrogen (white).

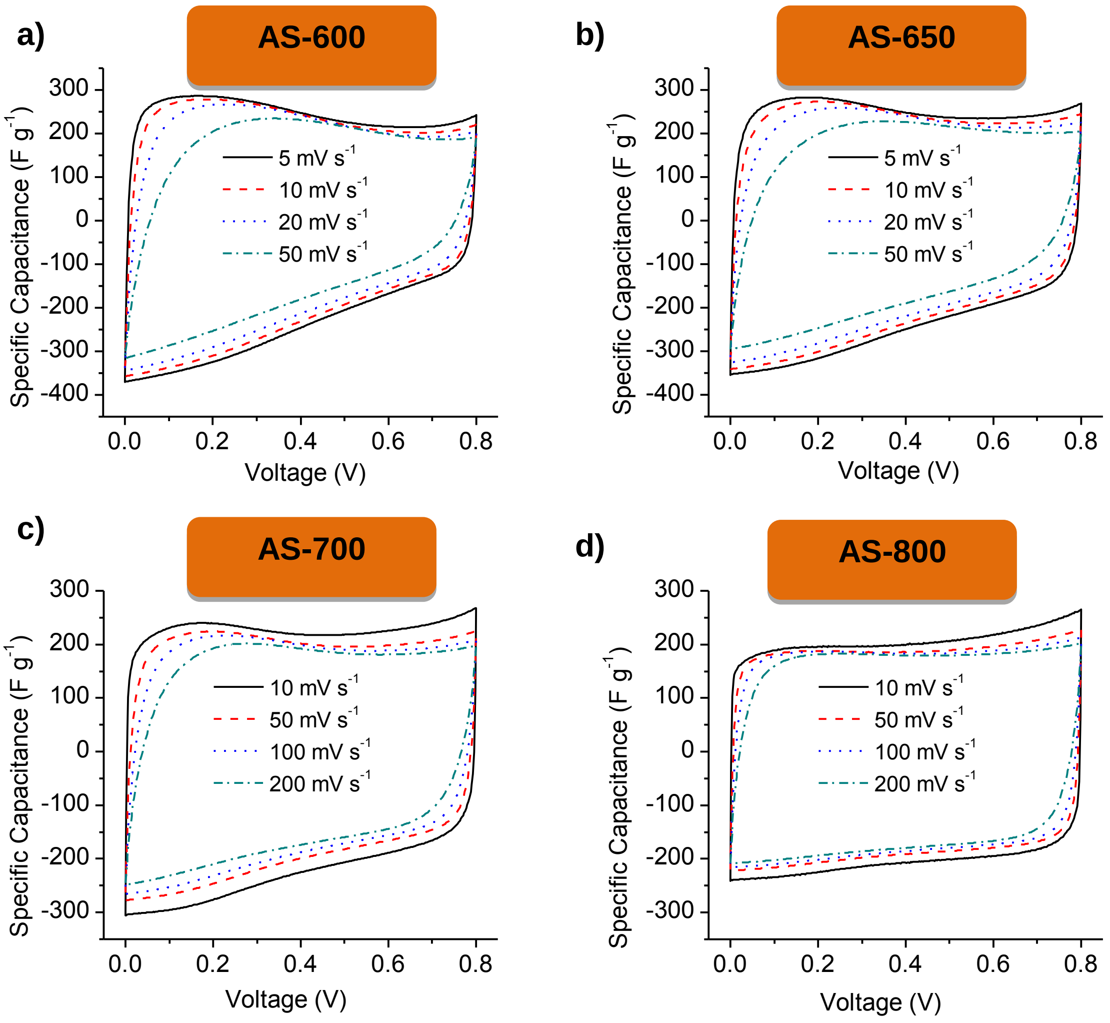

Figure S3. Cyclic voltammograms in a two-electrode cell configuration at different scan rates for (a) AS-600, (b) AS-650, (c) AS-700 and (d) AS-800. Electrolyte: 1 M H_2SO_4.

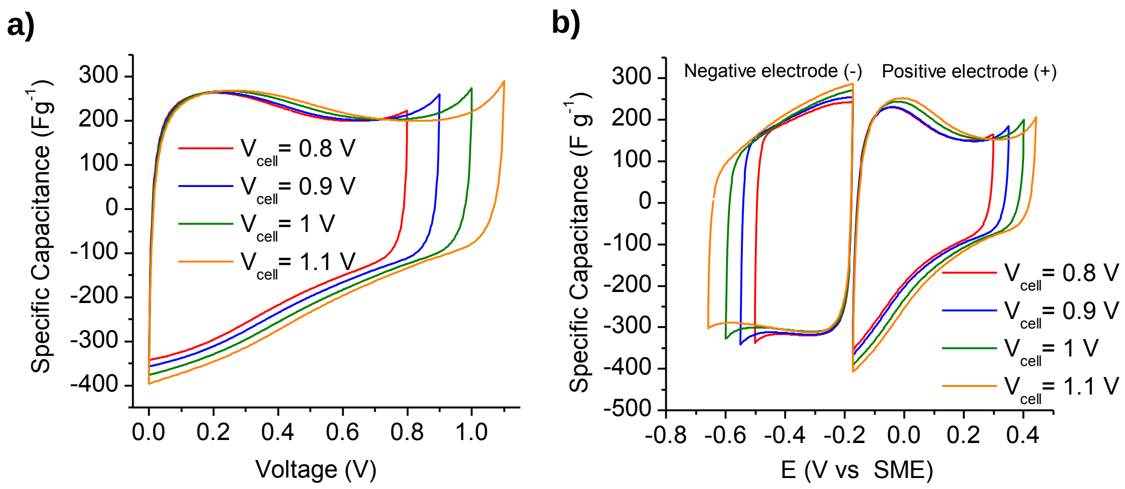

Figure S4. a) Enlargement of the voltage window examined by cyclic voltammetry for the AS-600 sample, and b) cyclic voltammograms for the positive and the negative electrode in a special two-electrode cell configuration provided with a SME reference electrode. Scan rate: at 5 mV s^{-1}, electrolyte: 1M H_2SO_4.

As can be seen in Figure S4b, the faradic redox reactions take place in the potential range of - 0.4 to 0.1 V *vs.* SME involving both the positive and the negative electrodes, which leads to the hump observed at cell voltages < 0.4-0.5 V in the CVs (Figure S4a) for the AS-600-based supercapacitor. This behavior is similar to that reported by Raymundo-Piñero *et. al* for N- and O-rich seaweed-derived carbons [1]. As a result of the larger pseudocapacitance contribution in the negative electrode, its working voltage window is smaller than that of the positive electrode (by around 0.13-0.15 V regardless of the cell voltage). In spite of this, the positive electrode does not undergo oxidation at high potentials, as can be seen in Figure S4b, and works below the water decomposition limit in 1M H_2SO_4 (*i.e.* around 0.6 V *vs.* SME). Neither does water reduction take place in the negative electrode, even though the

thermodynamic potential for water reduction is -0.62 V *vs.* SME in 1 M H_2SO_4. These results further support that carbon functionalization is an effective way to extend the working voltage range in acidic electrolyte [1,11]. Also worth mentioning is that, as measured in this special 2-electrode cell, the surface area-normalized capacitance of both the positive and the negative electrode experiences a 2-fold increase from 800 to 650 ºC, *i.e.* with increasing surface functionalization.

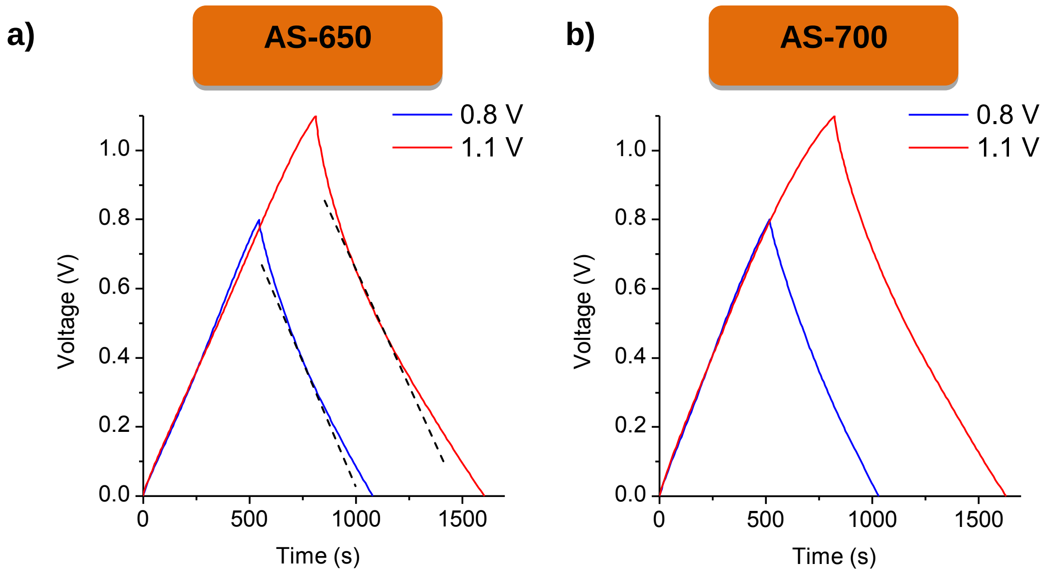

Figure S5. Enlargement of the voltage window as evaluated by CD at 200 mA g^{-1} for (a) AS-650 and (b) AS-700. Electrolyte: H_2SO_4.

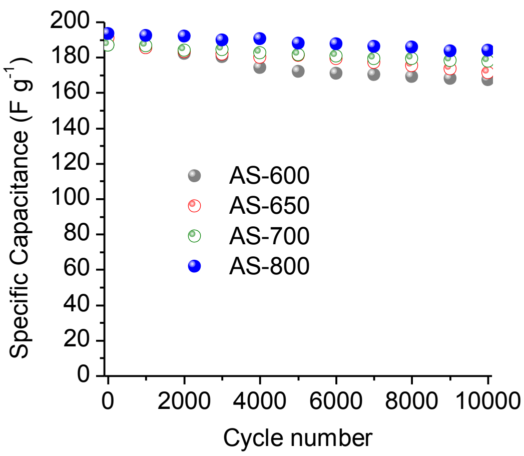

Figure S6. Fading of the specific capacitance of the microporous materials during long-term cycling at a constant current of 5 A g^{-1} in 1 M H_2SO_4 electrolyte.

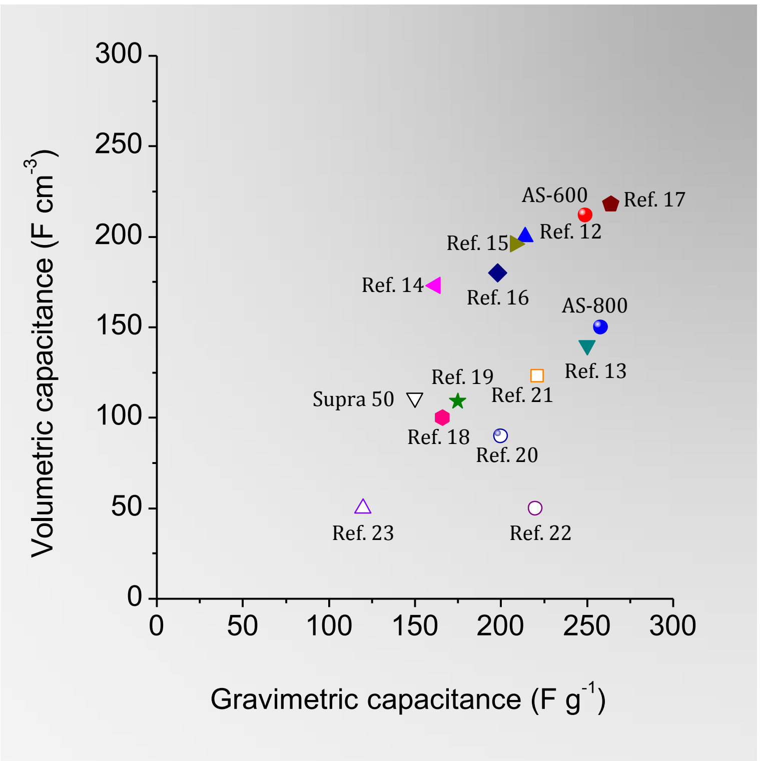

Figure S7. Comparison of the volumetric and gravimetric capacitances of AS-600 and AS-800 in an aqueous electrolyte using data reported in the literature [12-23].

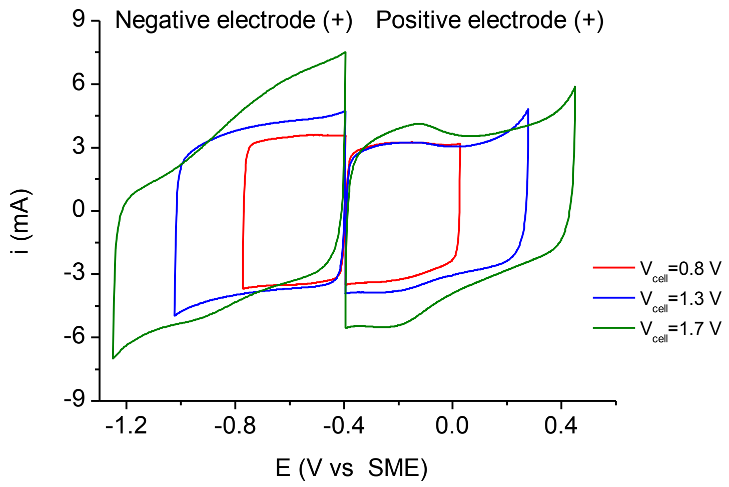

Figure S8. Cyclic voltammograms corresponding to the positive and the negative electrode in a special two-electrode cell configuration provided with a SME reference electrode for the different cell voltages. Scan rate: 5 mV s^{-1}, electrolyte: 1 M Li_2SO_4.

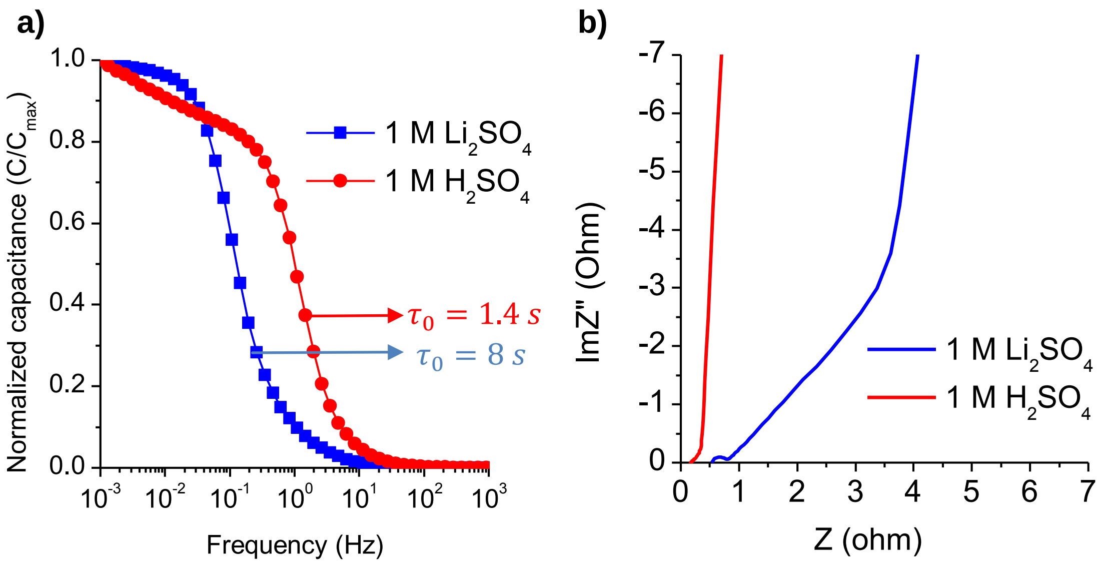

Figure S9. (a) Frequency response and (b) Nyquist plot for the AS-800 porous carbon. Electrolytes: 1 M Li_2SO_4 and 1 M H_2SO_4.

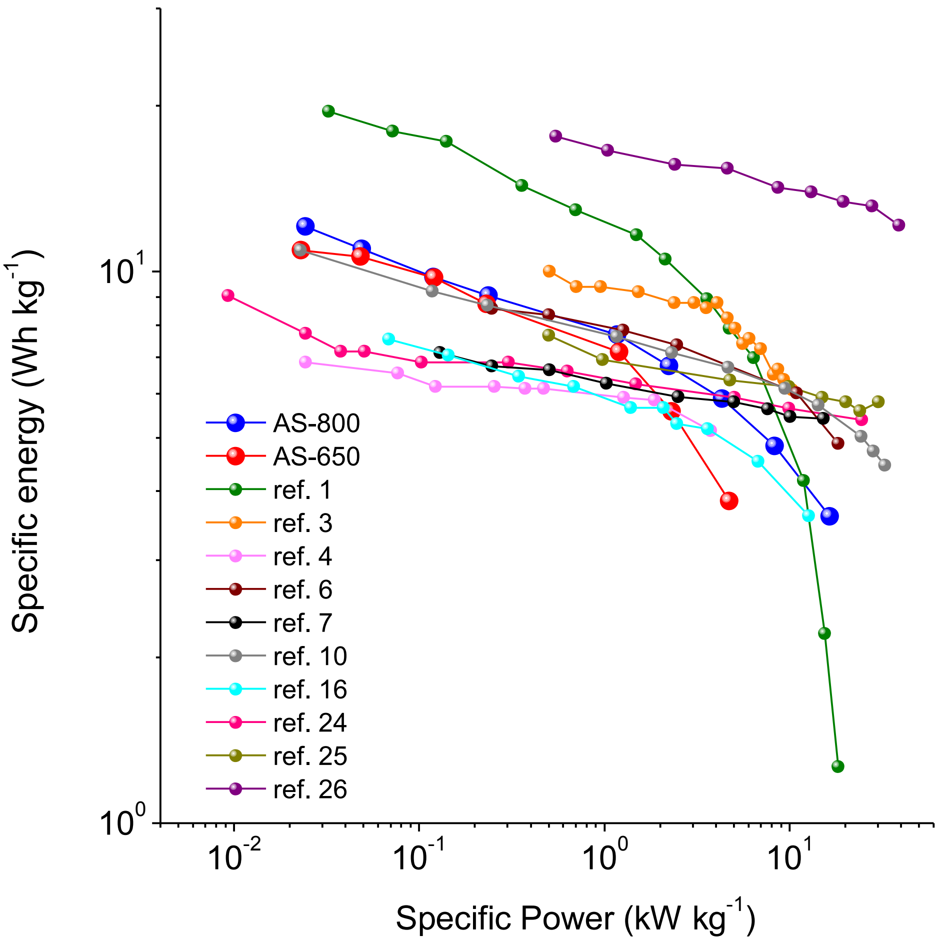

Figure S10. Ragone plot that compares the AS-800 and AS-650 materials developed in this work with data reported in the literature in H_2SO_4 or KOH aqueous electrolyte [1,3,4,6,7,10,16,24-26].

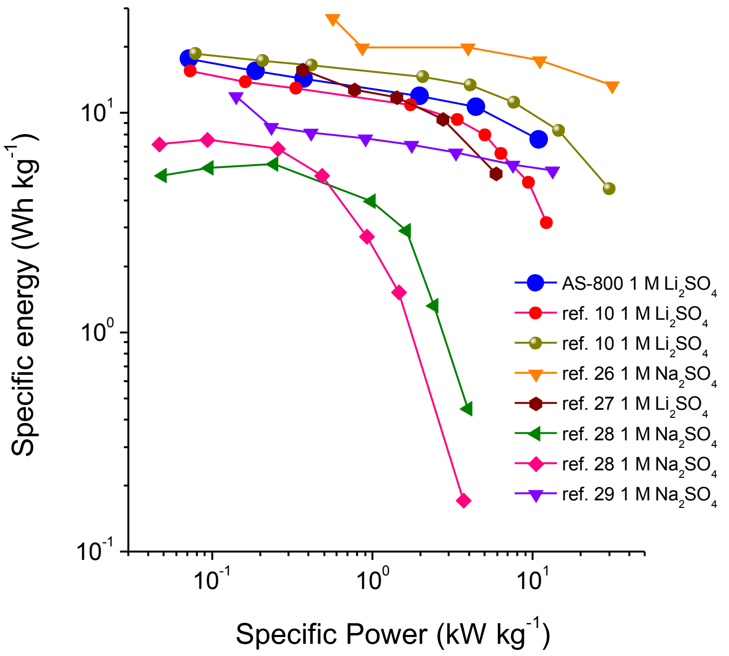

Figure S11. Ragone plot that compares the AS-800 material developed in this work with data reported in the literature in neutral aqueous electrolyte [10,26-29].

References

1 Raymundo-Piñero, E., Cadek, M. & Béguin, F. Tuning Carbon Materials for Supercapacitors by Direct Pyrolysis of Seaweeds. *Adv. Funct. Mater.* **19**, 1032-1039, (2009).

2 Jeong, H. M. *et al.* Nitrogen-Doped Graphene for High-Performance Ultracapacitors and the Importance of Nitrogen-Doped Sites at Basal Planes. *Nano Letters* **11**, 2472-2477, (2011).

3 Sun, L. *et al.* From coconut shell to porous graphene-like nanosheets for high-power supercapacitors. *J. Mater. Chem. A* **1**, 6462-6470, (2013).

4 Tao, Y. *et al.* Towards ultrahigh volumetric capacitance: graphene derived highly dense but porous carbons for supercapacitors. *Sci. Rep.* **3**, (2013).

5 Xu, B., Yue, S., Qiao, N., Chu, M. & Wei, G. Easy preparation of nitrogen-doped porous carbon nanospheres and their application in supercapacitors. *Materials Letters* **131**, 49-52, (2014).

6 Tan, Y. *et al.* Synthesis of Ultrathin Nitrogen-Doped Graphitic Carbon Nanocages as Advanced Electrode Materials for Supercapacitor. *ACS Applied Materials & Interfaces* **5**, 2241-2248, (2013).

7 Chen, L.-F. *et al.* Synthesis of Nitrogen-Doped Porous Carbon Nanofibers as an Efficient Electrode Material for Supercapacitors. *ACS Nano* **6**, 7092-7102, (2012).

8 Yun, Y. S. *et al.* Microporous Carbon Nanoplates from Regenerated Silk Proteins for Supercapacitors. *Adv. Mater.* **25**, 1993-1998, (2013).

9 Rufford, T. E., Hulicova-Jurcakova, D., Zhu, Z. & Lu, G. Q. Nanoporous carbon electrode from waste coffee beans for high performance supercapacitors. *Electrochem. Commun.* **10**, 1594-1597, (2008).

10 Fuertes, A. B. & Sevilla, M. Superior Capacitive Performance of Hydrochar-Based Porous Carbons in Aqueous Electrolytes. *ChemSusChem* **8**, 1049-1057, (2015).

11 Fuertes, A. B., Ferrero, G. A. & Sevilla, M. One-pot synthesis of microporous carbons highly enriched in nitrogen and their electrochemical performance. *J. Mater. Chem. A* **2**, 14439-14448, (2014).

12 Chaikittisilp, W. *et al.* Nanoporous carbons through direct carbonization of a zeolitic imidazolate framework for supercapacitor electrodes. *Chem. Commun.* **48**, 7259-7261, (2012).

13 Ruiz-Rosas, R. *et al.* Electrochemical Performance of Hierarchical Porous Carbon Materials Obtained from the Infiltration of Lignin into Zeolite Templates. *ChemSusChem* **7**, 1458-1467, (2014).

14 Xia, X., Shi, L., Liu, H., Yang, L. & He, Y. A facile production of microporous carbon spheres and their electrochemical performance in EDLC. *Journal of Physics and Chemistry of Solids* **73**, 385-390, (2012).

15 Lei, Z., Lu, L. & Zhao, X. S. The electrocapacitive properties of graphene oxide reduced by urea. *Energy Environ. Sci.* **5**, 6391-6399, (2012).

16 Raymundo-Piñero, E., Leroux, F. & Béguin, F. A High-Performance Carbon for Supercapacitors Obtained by Carbonization of a Seaweed Biopolymer. *Adv. Mater.* **18**, 1877-1882, (2006).

17 Xu, B. *et al.* Ultramicroporous carbon as electrode material for supercapacitors. *J. Power Sources* **228**, 193-197, (2013).

18 Lota, G., Lota, K. & Frackowiak, E. Nanotubes based composites rich in nitrogen for supercapacitor application. *Electrochemistry Communications* **9**, 1828-1832, (2007).

19 Yu, X., Wang, J.-g., Huang, Z.-H., Shen, W. & Kang, F. Ordered mesoporous carbon nanospheres as electrode materials for high-performance supercapacitors. *Electrochemistry Communications* **36**, 66-70, (2013).

20 Fernández, J. A., Arulepp, M., Leis, J., Stoeckli, F. & Centeno, T. A. EDLC performance of carbide-derived carbons in aprotic and acidic electrolytes. *Electrochim. Acta* **53**, 7111-7116, (2008).

21 Zapata-Benabithe, Z., Carrasco-Marín, F. & Moreno-Castilla, C. Preparation, surface characteristics, and electrochemical double-layer capacitance of KOH-activated carbon aerogels and their O- and N-doped derivatives. *J. Power Sources* **219**, 80-88, (2012).

22 Xia, K., Gao, Q., Jiang, J. & Hu, J. Hierarchical porous carbons with controlled micropores and mesopores for supercapacitor electrode materials. *Carbon* **46**, 1718-1726, (2008).

23 Luo, J., Jang, H. D. & Huang, J. Effect of Sheet Morphology on the Scalability of Graphene-Based Ultracapacitors. *ACS Nano* **7**, 1464-1471, (2013).

24 Wang, D.-W., Li, F., Liu, M., Lu, G. Q. & Cheng, H.-M. 3D Aperiodic Hierarchical Porous Graphitic Carbon Material for High-Rate Electrochemical Capacitive Energy Storage. *Angew. Chem. Int. Ed.* **47**, 373-376, (2008).

25 Zhang, L. & Shi, G. Preparation of Highly Conductive Graphene Hydrogels for Fabricating Supercapacitors with High Rate Capability. *The Journal of Physical Chemistry C* **115**, 17206-17212, (2011).

26 Wang, Q. *et al.* Interconnected porous and nitrogen-doped carbon network for supercapacitors with high rate capability and energy density. *Electrochim. Acta* **114**, 165-172, (2013).

27 Sun, X., Zhang, X., Zhang, H., Zhang, D. & Ma, Y. A comparative study of activated carbon-based symmetric supercapacitors in Li2SO4 and KOH aqueous electrolytes. *J. Solid State Electrochem.* **16**, 2597-2603, (2012).

28 Jiménez-Cordero, D., Heras, F., Gilarranz, M. A. & Raymundo-Piñero, E. Grape seed carbons for studying the influence of texture on supercapacitor behaviour in aqueous electrolytes. *Carbon* **71**, 127-138, (2014).

29 Wang, Q. *et al.* Template synthesis of hollow carbon spheres anchored on carbon nanotubes for high rate performance supercapacitors. *Carbon* **52**, 209-218, (2013).
